# Draft Genome Sequence of Acinetobacter sp. Strain YZS-X1-1, a Denitrifying Bacterium Isolated from Freshwater Pond Sludge in China

**DOI:** 10.1128/genomeA.01579-14

**Published:** 2015-02-12

**Authors:** Honggang Zhang, Xinzhi Li, Bingzhao Zhang, Chenli Liu

**Affiliations:** a Guangzhou Institute of Advanced Technology, Chinese Academy of Sciences, China; b Center for Synthetic Biology Engineering Research, Shenzhen Institutes of Advanced Technology, Chinese Academy of Sciences, Shenzhen, China

## Abstract

Acinetobacter sp. strain YZS-X1-1 was isolated from freshwater pond sludge in China. Here, we present the draft genome of strain YZS-X1-1, which consists of 3,278,660 bases, with a G+C content of 42.1%.

## GENOME ANNOUNCEMENT

Acinetobacter spp. have been isolated from clinical specimens (1) and environmental sources, such as activated sludge, wetlands, and forest soil (2), and they are characterized by nonmotile, Gram-negative, oxidase-negative, and catalase-positive organisms (1, 3, 4). Here, we present the draft genome sequence of an Acinetobacter bacterium, strain YZS-X1-1. The bacterium was isolated from freshwater pond sludge in China during our efforts to discover denitrifying bacteria for further environmental utilization. We preserved this bacterium on Luria-Bertani (LB) plates with broth supplemented at −80°C.

The genomic DNA of Acinetobacter sp. YZS-X1-1 was extracted and purified by a commercial genomic DNA isolation kit (Tiangen Corporation Ltd., Beijing, China). The genome sequence of Acinetobacter sp. YZS-X1-1 was detected by Shanghai Majorbio Bio-pharm Technology Co., Ltd. (Shanghai, China) with Solexa paired-end sequencing technology. The draft genome sequence of YZS-X1-1 consists of 79 contigs (length >200 bp; *N*_50_ contigs, 10), and a whole sequence size of 3,278,660 bp with an *N*_50_ contig size of 97,146 was determined with an Illumina/Solexa genome analyzer IIx (Illumina, San Diego, CA). The gaps among the scaffolds of the sequence were deleted by PCR amplification, followed by DNA sequencing or by custom primer walks. The mean G+C content was 42.1%, and the automatic gene annotation was performed using the NCBI Prokaryotic Genome Annotation Pipeline (PGAP) (http://www.ncbi.nlm.nih.gov/genomes/static/Pipeline.html). (5). The Acinetobacter sp. YZS-X1-1 contains 3,004 potential open reading frames (ORFs), including three rRNA operons and 75 tRNAs. The entire genome was assembled and recombined with SOAP*denovo* version 1.05 (6).

Acinetobacter sp. YZS-X1-1 demonstrated promising denitrifying ability to transform ammonia nitrogen in polluted water. Therefore, we particularly analyzed genes possibly responsible for the degradation of ammonia nitrogen, according to the annotation results. Many pathways (including those for signal transduction, protein export, and degradation) may be involved in the degradation of ammonia nitrogen. These genes may help this newly found bacterium make use of ammonia as its survival energy. Further research may lead to a better understanding of the bacterial denitrification of ammonia nitrogen.

### Nucleotide sequence accession numbers. 

This whole-genome shotgun project has been deposited at DDBJ/EMBL/GenBank under the accession no. JWHB00000000. The version described in this paper is the first version, accession no. JWHB01000000.
